# Roles of Polymorphonuclear Neutrophils in Ischemic Brain Injury and Post-Ischemic Brain Remodeling

**DOI:** 10.3389/fimmu.2021.825572

**Published:** 2022-01-11

**Authors:** Ayan Mohamud Yusuf, Nina Hagemann, Peter Ludewig, Matthias Gunzer, Dirk M. Hermann

**Affiliations:** ^1^ Department of Neurology, University Hospital Essen, Essen, Germany; ^2^ Center for Translational and Behavioral Neurosciences, University Hospital Essen, Essen, Germany; ^3^ Department of Neurology, University Medical Center Hamburg-Eppendorf, Hamburg, Germany; ^4^ Institute of Experimental Immunology and Imaging, University Hospital Essen, Essen, Germany; ^5^ Leibniz-Institut für Analytische Wissenschaften-ISAS-e.V., Dortmund, Germany

**Keywords:** focal cerebral ischemia, ischemic stroke, neuroinflammation, brain remodeling, polymorphonuclear neutrophils

## Abstract

Following ischemic stroke, polymorphonuclear neutrophils (PMNs) are rapidly recruited to the ischemic brain tissue and exacerbate stroke injury by release of reactive oxygen species (ROS), proteases and proinflammatory cytokines. PMNs may aggravate post-ischemic microvascular injury by obstruction of brain capillaries, contributing to reperfusion deficits in the stroke recovery phase. Thus, experimental studies which specifically depleted PMNs by delivery of anti-Ly6G antibodies or inhibited PMN brain entry, e.g., by CXC chemokine receptor 2 (CXCR2) or very late antigen-4 (VLA-4) blockade in the acute stroke phase consistently reduced neurological deficits and infarct volume. Although elevated PMN responses in peripheral blood are similarly predictive for large infarcts and poor stroke outcome in human stroke patients, randomized controlled clinical studies targeting PMN brain infiltration did not improve stroke outcome or even worsened outcome due to serious complications. More recent studies showed that PMNs have decisive roles in post-ischemic angiogenesis and brain remodeling, most likely by promoting extracellular matrix degradation, thereby amplifying recovery processes in the ischemic brain. In this minireview, recent findings regarding the roles of PMNs in ischemic brain injury and post-ischemic brain remodeling are summarized.

## Brain-Invading Polymorphonuclear Neutrophils (PMNs) as First Line Immune Response Contributing to Ischemic Damage

PMNs are first line immune invaders in the ischemic brain. PMN recruitment after experimental ischemic stroke is highly coordinated in a spatio-temporal way. PMNs accumulate within capillaries and venules of the ischemic brain territory within the first hour after ischemic stroke (1), followed by their extravasation into the perivascular space and tissue parenchyma, which gets significant in the first two days post-stroke (2–4). Depending on the severity of the ischemic episode, brain PMN peak numbers are reached between day 1 and day 3 (5). Transendothelial migration of PMN is mediated by adhesion molecules, e.g., of intercellular adhesion molecule 1 (ICAM-1) and platelet endothelial cell adhesion molecule-1 (PECAM-1), which exhibit an increased expression after induction of experimental ischemic stroke (6, 7). Infiltrating PMNs contribute to ischemic brain injury development, as revealed by studies, in which PMNs were depleted by delivery of anti-Ly6G antibodies or in which PMN accumulation was inhibited by anti-very late antigen-4 (VLA4) or anti-CXC chemokine receptor-2 (CXCR2) antibody delivery or mesenchymal stromal cell (MSC)-derived small extracellular vesicle (EV) administration (2, 3, 8). In each of these studies, the prevention of brain PMN brain entry was found to reduce ischemic brain injury and neurological deficits. These studies very clearly defined an injury-exacerbating role of PMNs in the acute stroke phase (9).

## PMN Hyperactivation in Peripheral Blood Is Associated With Secondary Brain Injury and Neurological Deterioration

Evidence in humans supports the idea that PMNs have detrimental consequences on stroke outcome. Hence, PMNs of ischemic stroke patients produce and release significant amounts of reactive oxygen species (ROS) and proteases, such as neutrophil elastase, as shown in peripheral blood within the first 6 hours post-stroke (10). Under ischemic conditions, peripheral blood PMNs are defined by a decreased surface abundance of L-selectin and increased expression of β2-integrins (10). This PMN hyperactivation facilitates PMN brain entry and was found to be associated with ischemic stroke progression in human patients (10, 11). The loss of microvascular integrity is an important component in this type of secondary brain injury. Thus, increased PMN responses, reflected by increased PMN numbers or, more specifically, a high neutrophil-to-lymphocyte ratio (NLR) in peripheral blood, are associated with an increased likelihood of intracerebral hemorrhage, death and poor neurological outcome at 3 months post-stroke, as assessed by the modified Rankin Scale (mRS) score in patients receiving tissue plasminogen activator (tPA)-induced thrombolysis (12).

## Microvascular Occlusion by PMN Stalls Exacerbates Secondary Brain Damage

A possible mechanism, *via* which PMNs in peripheral blood aggravate secondary ischemic brain damage are PMN stalls resulting in microvascular occlusions even under conditions of successful arterial reopening (13–15). In a murine thromboembolic stroke model, intravenous thrombolysis initiated 30 minutes after embolization achieved the successful resolution of blood clots in the M2 segment of the middle cerebral artery (14). Yet, as many as ~35% of capillaries in the evolving infarct core and ~15% of capillaries in the surrounding infarct periphery exhibited microvascular occlusions compromising brain tissue recovery (14). Strikingly, PMNs made up as many as 67% of capillary stalls in the infarct core and 54% of the capillary stalls in the infarct periphery (14). Delivery of anti-Ly6G antibody prior to stroke significantly reduced capillary obstructions and hemorrhagic transformation, and improved tissue perfusion and sensorimotor function after stroke (14).

## Randomized Controlled Clinical Trials Failed to Show a Beneficial Effect of PMN Inhibition Strategies

Despite numerous studies describing a deleterious role of PMNs post-stroke, randomized controlled clinical trials aiming at reducing PMN brain accumulation did not aid stroke recovery in ischemic stroke patients. Thus, the delivery of a murine monoclonal antibody directed against intercellular adhesion molecule-1 (ICAM1) within 6 hours of symptom onset aggravated neurological recovery assessed by the mRS score, increased stroke mortality and increased infection susceptibility (16). Besides, the administration of the glycoprotein UK279,276, a CD11/CD18 integrin antagonist, within 6 hours of symptom onset did not improve stroke outcome in stroke patients receiving recombinant tPA-induced thrombolysis, as compared to patients receiving tPA alone (17). Functional deficits were also not alleviated after treatment with natalizumab, which is a monoclonal antibody targeting VLA-4 (18, 19). Similarly, the delivery of a humanized monoclonal antibody against CD11/CD18 did not beneficially influence stroke outcome (20). These clinical findings necessitate a more differentiated assessment of the role of PMNs in the ischemic brain. In fact, previous studies largely neglected the post-acute stroke phase in which PMNs have diverse roles.

## Neutrophil Extracellular Traps (NETs) Compromise Post-Ischemic Reperfusion and Microvascular Integrity

Upon injury, PMNs release NETs, which are composed of PMN DNA, histones and granule components such as elastase and cathepsin G (21). These NETs have recently been reported to impair microvascular integrity after stroke induction in mice by electrocoagulation of the middle cerebral artery (MCA) (22). In this study, NETs were identified based on citrullinated histone H3 (H3Cit) abundance, enrichment of Sytox green-labeled DNA fibers and the presence of the PMN marker Ly6G (22). Disruption of NET formation by means of DNase-1 injection increased blood-brain barrier integrity and increased microvascular survival in the peri-infarct cortex (22). The aggravation of microvascular injury by NETs may at least partly be attributed to the impaired lysis of blood clots, which abundantly contain citrullinated histones (23). *Ex vivo*, the treatment of blood clots obtained from ischemic stroke patients with DNAse-1 significantly increased tPA-induced thrombolysis in comparison to tPA alone (23).

## N2 Polarization Might Confer a Neuroprotective and Restorative Phenotype

**Figure 1:**
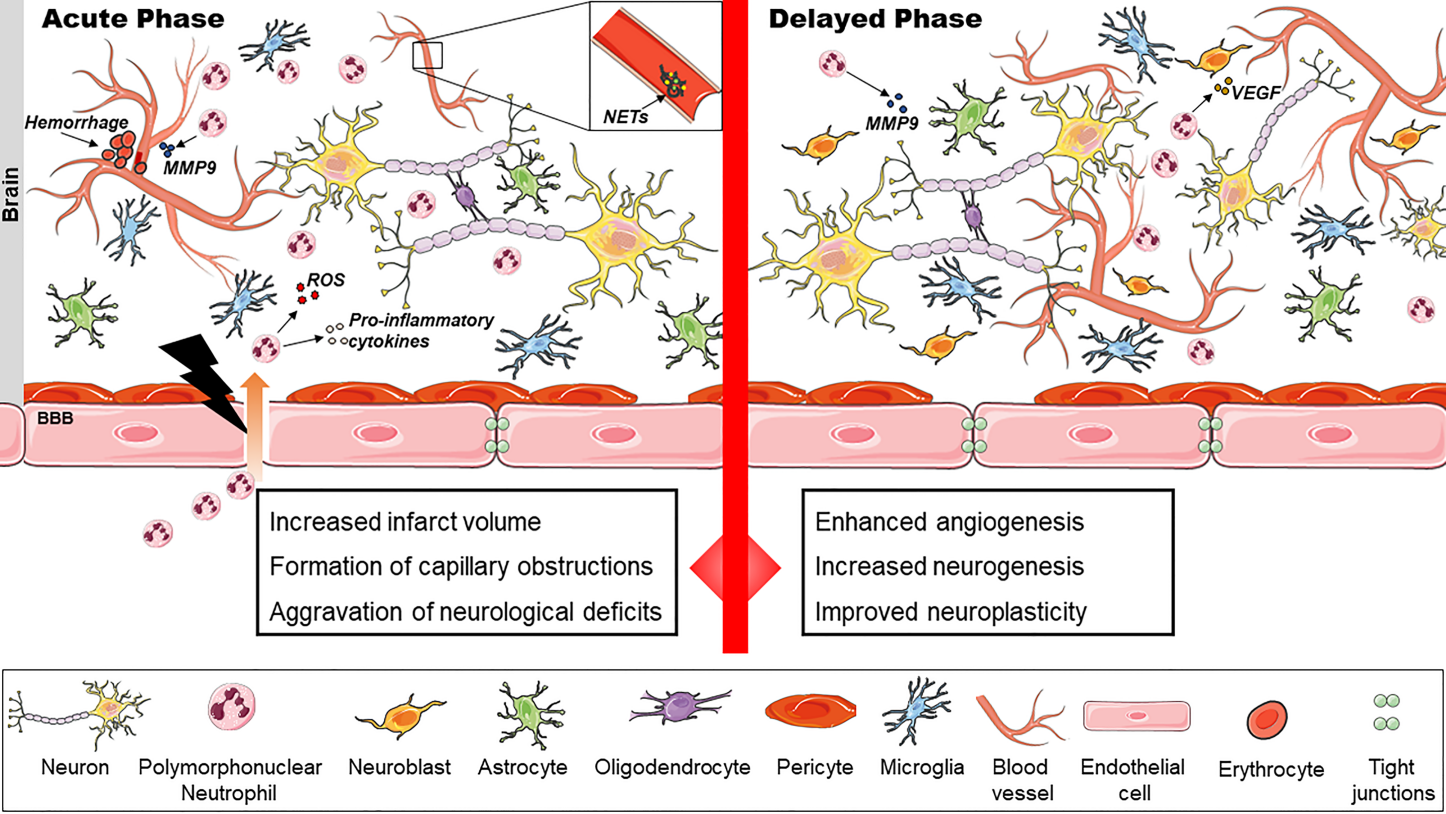
The dual role of polymorphonuclear neutrophils (PMNs) in ischemic stroke. PMNs exacerbate stroke injury in the acute phase by release of reactive oxygen species (ROS) and inflammatory cytokines. Intracerebral hemorrhages may result. Neutrophil extracellular traps (NET) contribute to capillary reperfusion disturbances. In the delayed phase, PMNs enhance cerebral angiogenesis, neurogenesis and neuroplasticity presumably by VEGF release, extracellular matrix (ECM) degradation and remodeling.

PMNs are a heterogeneous group of leukocytes, which might obtain proinflammatory or anti-inflammatory properties depending on the tissue microenvironment (24). The former PMNs, which are widely referred to as N1 phenotype, contain high levels of proinflammatory cytokines, ROS, Fas (CD95) and ICAM-1 (24–26) (**Table 1**). These PMNs vigorously recruit and activate CD8^+^ T cells in tissues (24). On the other hand, so-called N2 PMNs are characterized by their high content of arginase, vascular endothelial growth factor (VEGF), CC-chemokine-ligand-2 (CCL2) and CC-chemokine-ligand-5 (CCL5) (24, 27, 29) (**Table 1**). These PMNs may promote tissue survival and remodeling in the ischemic brain (**Figure 1**). Indeed, treatment with rosiglitazone, an activator of peroxisome proliferator-activated receptor-γ (PPARγ), increased the number of brain-infiltrating PMNs with N2 phenotype at 24 hours after permanent middle cerebral artery occlusion (MCAO) in mice (31). Infarct volume was reduced by rosiglitazone; this neuroprotection was abrogated after PMN depletion induced by anti–PMN antibody delivery (31). In another study, increased numbers of brain-infiltrating PMNs with N2 phenotype were noted in ischemic brains of toll-like receptor-4 deficient compared to wildtype mice after MCAO (32). In this study, PMN accumulation was inversely correlated with infarct volume (32), suggestive of a neuroprotective role of N2 PMNs.

**Table 1:** Neutrophil polarization.

Phenotype	Effector molecule
**N1 neutrophils**	ROS (25) TNF-α (25) ICAM-1 (26) Fas (24)
**N2 neutrophils**	MMP9 (27, 28) VEGF (27) Arginase (24) CCL2, CCL5 (29)

Depending on the microenvironment, neutrophils adapt an N1 or N2 phenotype which differ in their effector molecules. Currently, N1 and N2 neutrophils are not distinguishable by distinct surface markers (30). Yet, their biological actions greatly differ from each other, as this table shows. Morphologically, N1 neutrophils are characterized by hypersegmented nuclei (24).

## PMNs Are Indispensable for Brain Remodeling in the Post-Acute Stroke Phase

MSC-derived EVs potently promote angiogenesis in the ischemic brain (33), specifically when these EVs are obtained from MSCs cultured under hypoxic conditions (33, 34). This effect depends on the presence of PMNs, as recently shown by us. Thus, PMN depletion by anti-Ly6G antibody delivery at 24 hours after MCAO abolished the angiogenic effects of MSC-EVs (34). As an important source of VEGF, PMNs directly stimulate angiogenesis (27). Endothelial cells in turn release growth factors which protect the brain, promote neuroplasticity and induce neurogenesis (35). Newly formed blood vessels support the migration of neuronal progenitor cells from the subventricular zone to the ischemic brain region (36). Inhibition of vascular endothelial growth factor receptor-2 (VEGFR2) impairs the production of growth factors, namely of brain-derived neurotrophic factor (BDNF) (37). Besides, PMNs have important roles in extracellular matrix (ECM) remodeling by production and secretion of matrix metalloproteinase-9 (MMP9) (28). In rats exposed to MCAO, MMP9 formation was markedly reduced in response to neutropenia induced by the chemotherapeutic vinblastine (38). MMP9 has diverse effects on ischemic injury and brain tissue remodeling, depending on the time-point post-ischemic injury. Post mortem studies in humans revealed robust MMP9 expression in the perivascular space of demarcating brain infarcts (39). PMN-derived MMP9 was localized in proximity to leaky microvessels exhibiting basal lamina disruption (40). Hence, MMP9 expression was found to predispose to hemorrhagic transformation in the acute stroke phase (41). In the post-acute stroke phase, the risk of brain hemorrhage formation has already vanished, and ECM remodeling appears to be a prerequisite for infarct removal and microvascular sprouting (42). In rats exposed to MCAO, delayed MMP inhibition 7 days after MCAO reduced the expression of the neuroplasticity marker early growth response protein-1 (Egr1), decreased the number of RECA-1^+^ endothelial cells, increased sensorimotor deficits and increased ischemic injury, while acute MMP inhibition on day 1 reduced infarct volume (43). In mice, lentiviral MMP9 overexpression initiated on day 7 after MCAO facilitated glial scar resolution, increased microvessel numbers and endogenous neurogenesis and increased the expression of synaptic plasticity markers in the peri-infarct brain (44). The delivery of a vascular endothelial growth factor receptor-2 (VEGFR2) inhibitor or a MMP9 inhibitor reversed these effects (44). Taken together, these results emphasize the close interdependence of post-ischemic angiogenesis, neurogenesis and synaptic plasticity. All three processes rely on the coordinated reshaping of the ECM.

## Conclusions

There is robust evidence that PMNs aggravate ischemic brain injury, neurological deficits and hemorrhagic transformation in the acute stroke phase. However, it also becomes clear that PMNs and PMN-derived extracellular matrix proteases are indispensable for successful brain remodeling and neurological recovery in the post-acute stroke phase, perhaps mediated by a restorative N2 phenotype. Future studies will have to dissect the diverse roles of PMNs, including proinflammatory N1 and restorative N2 phenotypes, define their temporospatial significance and their consequences for ischemic injury and stroke recovery. Only by a thorough understanding of these partly opposing actions, we will be able to define pathophysiological processes *via* which PMNs can therapeutically be targeted.

## Author Contributions

AM and DH designed and wrote the article. NH, PL, and MG critically revised the article. All authors approved the submitted version.

## Funding

Supported by the German Research Foundation (FOR-2879 project 405358801 to DH, MG, and PL and project 389030878 to DH and MG) and Hertie-Stiftung (Hertie Academy of Clinical Neuroscience to PL).

## Conflict of Interest

The authors declare that the research was conducted in the absence of any commercial or financial relationships that could be construed as a potential conflict of interest.

## Publisher’s Note

All claims expressed in this article are solely those of the authors and do not necessarily represent those of their affiliated organizations, or those of the publisher, the editors and the reviewers. Any product that may be evaluated in this article, or claim that may be made by its manufacturer, is not guaranteed or endorsed by the publisher.
